# The Relationships Between Serum DHEA-S and AMH Levels in Infertile Women: A Retrospective Cross-Sectional Study

**DOI:** 10.3390/jcm10061211

**Published:** 2021-03-15

**Authors:** Li-Te Lin, Kuan-Hao Tsui

**Affiliations:** 1 Department of Obstetrics and Gynecology, Kaohsiung Veterans General Hospital, Kaohsiung 81362, Taiwan; khtsui@vghks.gov.tw; 2 Institute of BioPharmaceutical Sciences, National Sun Yat-Sen University, Kaohsiung 80424, Taiwan; 3 Department of Obstetrics and Gynecology, National Yang-Ming University School of Medicine, Taipei 112, Taiwan

**Keywords:** DHEA-S, dehydroepiandrosterone, anti-Mullerian hormone (AMH), ovarian reserve, infertile women

## Abstract

The relationship between serum dehydroepiandrosterone sulphate (DHEA-S) and anti-Mullerian hormone (AMH) levels has not been fully established. Therefore, we performed a large-scale cross-sectional study to investigate the association between serum DHEA-S and AMH levels. The study included a total of 2155 infertile women aged 20 to 46 years who were divided into four quartile groups (Q1 to Q4) based on serum DHEA-S levels. We found that there was a weak positive association between serum DHEA-S and AMH levels in infertile women (r = 0.190, *p* < 0.001). After adjusting for potential confounders, serum DHEA-S levels positively correlated with serum AMH levels in infertile women (β = 0.103, *p* < 0.001). Infertile women in the highest DHEA-S quartile category (Q4) showed significantly higher serum AMH levels (*p* < 0.001) compared with women in the lowest DHEA-S quartile category (Q1). The serum AMH levels significantly increased across increasing DHEA-S quartile categories in infertile women (*p* = 0.014) using generalized linear models after adjustment for potential confounders. Our data show that serum DHEA-S levels are positively associated with serum AMH levels.

## 1. Introduction

Anti-Mullerian hormone (AMH), a member of the transforming growth factor beta family, is specifically secreted by granulosa cells of small growing follicles [1]. Follicles release increased amounts of AMH until the follicles grow to 8 mm, and then dominant follicle selection is initiated by follicle-stimulating hormone (FSH). When follicles grow to over 8 mm during the FSH-dependent stage, AMH expression rapidly declines until it is absent [2]. Serum AMH levels are strongly and positively associated with the number of growing follicles and can serve as a serum marker for functional ovarian reserve [3]. However, serum AMH levels negatively correlate with age in adult women. After 25 years of age, serum AMH levels begin to drop steadily to undetectable levels at menopause [4,5]. Since serum AMH levels diminish yearly with age, it could be a possible predictor of natural fecundability in women [6]. Furthermore, serum AMH levels have been suggested to be an accurate predictor of ovarian responses to controlled ovarian hyperstimulation in women undergoing in vitro fertilization (IVF) cycles [7,8]. Some factors, such as the use of oral contraception and obesity, show potential negative correlations with serum AMH levels; however, some factors, such as vitamin D deficiency and BRCA mutations, demonstrate controversial results [3,9].

Dehydroepiandrosterone (DHEA), a 19 carbon endogenous steroid hormone, is naturally synthesized in our body through the cholesterol to pregnenolone pathway [10]. DHEA is mainly produced by the zona reticularis of the adrenal gland and is subsequently converted to its sulfated conjugate, dehydroepiandrosterone sulphate (DHEA-S), by the enzyme sulphotransferase [10,11]. DHEA and DHEA-S are the most abundant steroid hormones in humans, which decline steadily with age [11]. DHEA and DHEA-S production increases during adrenarche in children from the age of 6 to 8 years and reaches a peak at around the age of 20 years. At the age of 25, DHEA and DHEA-S secretion begins to decline and reaches very low or negligible levels after age 70 [11,12]. DHEA-S readily converts back to DHEA through hydrolysis by a sulphatase in peripheral target tissues and serves as a precursor to androgens and estrogens [12,13]. DHEA-S can enter the ovarian follicle and is a key source of ovarian testosterone, which plays an important role in promoting follicular development [14,15]. Meta-analyses have shown that oral administration of DHEA supplementation was associated with an increase in serum testosterone [16] and AMH levels [17].

Considering the abovementioned information, we hypothesized that there is a positive association between serum DHEA-S and AMH levels. Since there are limited studies that have examined this relationship, we performed a large cross-sectional study in infertile women to explore the correlation between serum DHEA-S and AMH levels.

## 2. Materials and Methods

### 2.1. Study Design and Participants

In this retrospective cross-sectional study, we identified infertile women based on the International Classification of Diseases, Ninth Revision, Clinical Modification (ICD-9-CM), code 628, from the clinical database at the Kaohsiung Veterans General Hospital. Among the infertile women identified by the ICD-9-CM code, in order to avoid any potential misclassifications, only subjects who had received a complete infertility examination at the Reproductive Center of Kaohsiung Veterans General Hospital were selected. A total of 2476 infertile women were identified from May 2013 to March 2020. Among them, subjects who had experienced repeated examinations, who were aged <20 or >46 years, who had been diagnosed with primary ovarian insufficiency, and who had androgen supplementation or hormonal therapy during the previous 3 months were excluded from this study. A total of 2155 infertile women were finally included in the study. The study protocol was approved by the institutional review board at the Kaohsiung Veterans General Hospital, with the identifier KSVGH20-CT11-03, and conformed to the “Declaration of Helsinki for Medical Research involving Human Subjects”. The requirement for informed consent was waived by the institutional review board of the Kaohsiung Veterans General Hospital.

### 2.2. Biochemical Measurements

Blood levels including AMH, testosterone (T), DHEA-S, FSH, luteinizing hormone (LH), estradiol, thyroid-stimulating hormone (TSH), prolactin, and 25(OH)Vitamin D were checked in our routine infertility examinations. Serum AMH values were measured using a Gen II Beckman Coulter AMH ELISA kit (Beckman Coulter, Marseille, France). The lower limit of detection was 0.02 ng/mL. The intra-assay and inter-assay coefficients of variation were 3.0% and 7.0%, respectively. In this study, diminished ovarian reserve (DOR) was defined as serum AMH levels <1.2 ng/mL based on the POSEIDON criteria [18], and serum AMH levels ≥5.0 ng/mL, modified from the revised Rotterdam criteria [19], were considered to be excess ovarian reserve (EOR).

Serum T, DHEA-S, FSH, LH, estradiol, TSH, prolactin, and 25(OH)Vitamin D levels were measured using a chemiluminescent microparticle immunoassay (CMIA) on the ARCHITECT iSystem (Abbott, Longford, Ireland or Abbott, Wiesbaden, Germany). The coefficient of variation of all the analyses were consistently from 3 to 10%. Blood samples were collected on the 2nd or 3rd days of the menstrual cycle. The reference intervals in the follicular phase are as follows: T, 0.14–0.53 ng/mL; DHEA-S, 35.0–430.0 μg/dL; FSH, 4–13 mIU/mL; LH, 1–18 mIU/mL; estradiol, 39–189 pg/mL; TSH, 0.35–4.94 μIU/mL; prolactin, 1.39–24.20 ng/mL; and 25(OH)Vitamin D, 30~100 ng/mL.

### 2.3. Statistical Analysis

The normality of the distribution was examined using the Kolmogorov–Smirnov test. Continuous variables are presented as the mean ± standard deviation (SD). The association between serum DHEA-S and AMH levels was examined using Pearson correlation coefficient and multivariate linear regression. In the multivariate linear regression, potential confounders including age, body mass index (BMI), FSH, and prolactin were adjusted. Then, subjects were categorized into four quartile groups based on serum DHEA-S levels. The characteristics of subjects among DHEA-S quartile categories were compared using analysis of variance (ANOVA) for continuous variables. The AMH trends across increasing DHEA-S quartile categories were confirmed by assessing *p*-values for trends using generalized linear models after adjustment for potential confounders. All analyses were performed using statistical software, Statistical Package for Social Sciences (SPSS) version 20.0 (Chicago, IL, USA). All statistical tests used a two-tailed α of 0.05. Statistical significance was defined as *p* < 0.05.

## 3. Results

Table 1 presents the characteristics of the study population. For the study population (*n* = 2155), the mean age was 35.1 ± 4.7 years (range 21–46 years), and the average BMI was 22.4 ± 3.8 kg/m^2^ (range 14.7–40.8 kg/m^2^). The mean serum DHEA-S level was 240.7 ± 113.6 μg/dL (range 10.1–1122.0 μg/dL) and the average serum AMH level was 4.0 ± 4.0 ng/mL (range 0.02–50.40 ng/mL). In the group of women <35 years (*n* = 972), the mean serum DHEA-S level was 262.7 ± 107.6 μg/dL, and the mean serum AMH level was 5.2 ± 4.5 ng/mL. In women ≥ 35 years (*n* = 1183), the average serum DHEA-S level was 222.5 ± 115.3 μg/dL, and the average serum AMH level was 2.9 ± 3.1 ng/mL.

**Figure 1:**
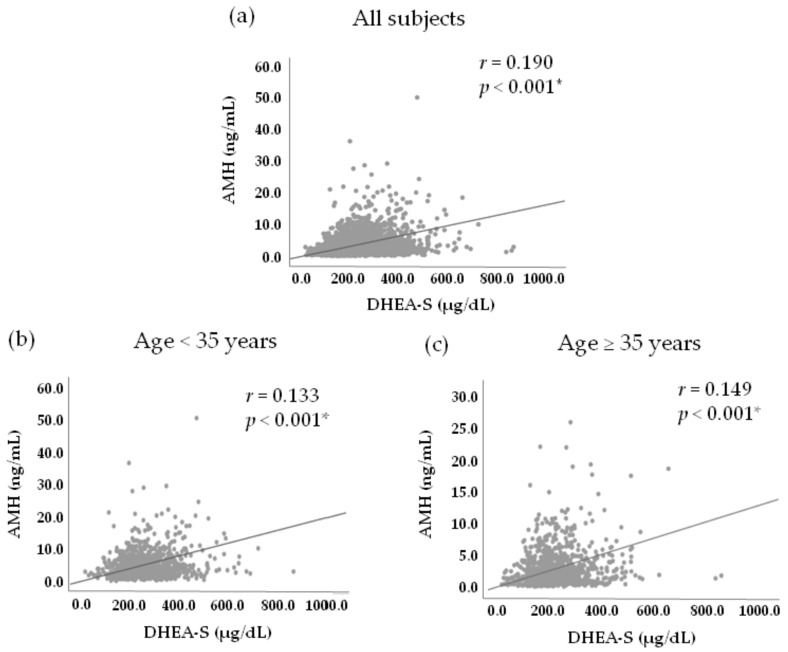
Correlations between serum DHEA-S and AMH levels. A weak positive correlation was observed for (**a**) all subjects; (**b**) age < 35 years; (**c**) age ≥ 35 years. * The *p*-value was generated using Pearson correlation coefficient.

As shown in Figure 1, considering all subjects, a weak positive correlation was observed between serum DHEA-S and AMH levels for all subjects (*r* = 0.190, *p*-value < 0.001), for women aged < 35 (*r* = 0.133, *p*-value < 0.001), and for women aged ≥ 35 (*r* = 0.149, *p*-value < 0.001).

Multiple linear regression analysis was performed to assess the relationship between serum DHEA-S and AMH levels after adjusting for potential confounders (age, BMI, FSH, and prolactin). Serum DHEA-S values were positively associated with serum AMH levels for all subjects (β = 0.103, *p*-value <0.001), for women aged < 35 (β = 0.113, *p*-value = 0.004), and for women aged ≥ 35 years (β = 0.091, *p*-value = 0.009), as shown in Table 2.

Next, the subjects were categorized into four quartile groups (Q1 to Q4) based on serum DHEA-S levels (Table 3). AMH and T were positively associated with the DHEA-S quartile category, whereas age and FSH decreased as the DHEA-S quartile category increased from Q1 to Q4 (all *p*-values < 0.001). Furthermore, as compared with women in the lowest DHEA-S quartile category (Q1), those in the highest DHEA-S quartile category (Q4) demonstrated significantly higher serum AMH values.

**Figure 2:**
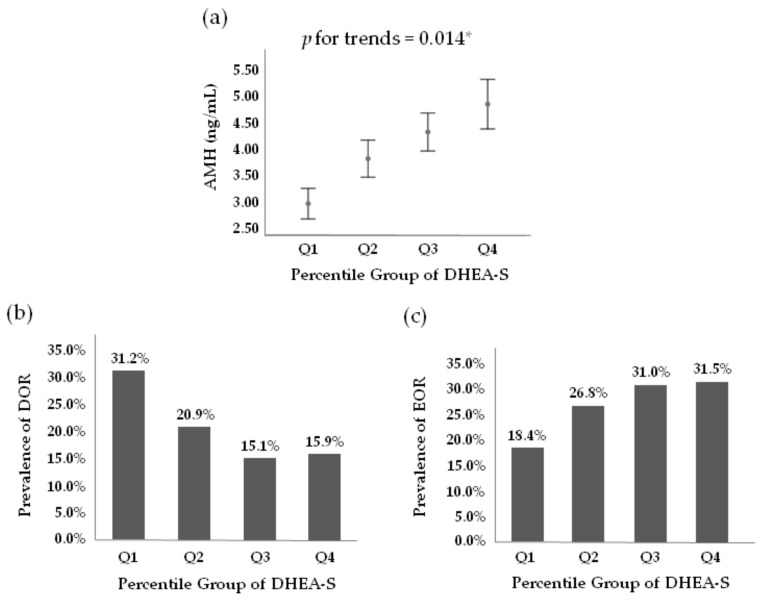
Correlations between serum DHEA-S quartile categories and AMH levels. (**a**) Serum AMH levels significantly increased across the increasing DHEA-S quartile categories. * The *p*-value for the trends was generated using generalized linear models after adjustment for potential confounders including age, BMI, FSH, and prolactin. Error bars represent 95% confidence interval of serum AMH levels; (**b**) prevalence of diminished ovarian reserve (DOR) according to serum DHEA-S quartile categories. DOR was defined as serum AMH levels < 1.2 ng/mL; (**c**) prevalence of excess ovarian reserve (EOR) based on serum DHEA-S quartile categories. EOR was defined as serum AMH values ≥ 5.0 ng/mL. AMH, anti-Mullerian hormone; DHEA-S, dehydroepiandrosterone sulphate; Q, quartile category.

Generalized linear models were conducted to evaluate the correlation of serum DHEA-S quartile categories with serum AMH levels after adjusting for potential confounding factors including age, BMI, FSH, and prolactin. In the multivariate adjustment model, shown in Figure 2a, the serum AMH levels significantly increased across increasing DHEA-S quartile categories (*p*-value = 0.014). The overall proportions of subjects who met the criteria for DOR (AMH < 1.2 ng/mL) and EOR (AMH ≥ 5.0 ng/mL) were 20.5% (345/1681) and 27.1% (456/1681), respectively. The prevalence of DOR among subjects in Q1, Q2, Q3, and Q4 were 31.2% (122/391), 20.9% (89/425), 15.1% (66/436), and 15.9% (68/429), respectively (Figure 2b). The prevalence of EOR among subjects in Q1, Q2, Q3, and Q4 were 18.4% (72/391), 26.8% (114/425), 31.0% (135/436), and 31.5% (135/429), respectively (Figure 2c).

Table 4 shows the age-dependent distribution of serum DHEA-S levels for all subjects (*n* = 2061). The median of serum DHEA-S levels for all subjects was 221.0 μg/dL; the medians of serum DHEA-S levels for age groups 20–25, 26–30, 31–35, 36–40, and 41–46 years were 272.3, 264.5, 234.2, 206.1, and 181.4 μg/dL, respectively.

## 4. Discussion

To the best of our knowledge, the current study is the largest cross-sectional study to examine the relationship between serum DHEA-S and AMH levels in infertile women. This study demonstrated that higher serum DHEA-S levels correlated with higher serum AMH levels in infertile women after adjusting for potential confounding factors. Consistently, DHEA-S quartile categories were positively associated with serum AMH levels in infertile women after adjustment for potential confounders.

DHEA acts as an indirect intermediate for androgens and estrogens. A meta-analysis showed that testosterone levels were significantly increased after DHEA administration, especially in healthy young women with higher DHEA dosage (>50 mg/day) and intervention duration of ≤12 weeks [16]. Consistently, this study revealed that serum T levels were positively related to the DHEA-S quartile category (Table 3). Moreover, Hu et al. showed that DHEA supplementation could enhance the expression of the androgen receptor (AR) in preovulatory granulosa cells [20]. Via the AR, androgens boost the expression of FSH receptor and stimulate FSH-mediated follicle development and granulosa cell proliferation [21,22,23]. The AMH secretion increased after FSH-mediated granulosa cell proliferation [24]. Thus, Dewailly et al. suggested that androgens increase AMH production through enhancement of FSH action [25]. Indeed, some studies have shown a positive correlation between serum T and AMH levels [26,27]. A meta-analysis of 24 randomized controlled trials involving 1429 participants revealed that serum insulin-like growth factor-1 (IGF-1) levels were significantly increased following DHEA supplementation [28]. It has been suggested that IGF-1 may also enhance FSH-stimulated follicle growth and granulosa cell differentiation [29,30,31,32]. Additionally, IGF-1 has been demonstrated to be able to promote primordial follicle activation [33]. Follicular fluid IGF-I levels, which positively correlate with serum IGF-I levels, have been reported to be related to ovarian reserve [34]. Taken together, DHEA supplementation may enhance AMH production, probably through increased levels of testosterone and IGF-1, which indirectly supports the results of the present study. However, further studies are still mandatory to confirm the relationship between serum DHEA-S and AMH levels and clarify the possible pathophysiology within the relationship.

There are conflicting results regarding the effect of DHEA supplementation on serum AMH levels. Several studies have shown that serum AMH levels significantly improved after DHEA supplementation [35,36,37,38,39]. A retrospective cross-sectional and longitudinal analysis of 120 women with DOR conducted by Gleicher et al. revealed that AMH levels significantly increased after DHEA supplementation over time and were more pronounced in women under age 38 years [35]. Yilmaz et al. performed a prospective study of 41 women with DOR and showed that there were significant improvements in AMH levels (0.32 ± 0.29 vs. 0.75 ± 0.70 ng/mL) before and after DHEA supplementation [36]. In a prospective study of 50 poor ovarian responders (PORs), Zangmo et al. demonstrated a significant increase in serum AMH levels from 1.20 ± 0.40 to 2.67 ± 0.80 ng/mL in women ≤35 years and from 0.90 ± 0.30 to 2.30 ± 0.80 ng/mL in women ≥36 years following DHEA treatment [38]. A meta-analysis also revealed that DHEA pretreatment resulted in significantly higher serum AMH levels [17]. However, some studies have demonstrated no change of serum AMH levels following DHEA supplementation [20,40,41]. A randomized controlled pilot study of 32 PORs conducted by Yeung et al. showed that no statistically significant differences in the ovarian reserve markers (AFC, AMH, or FSH) were found between the DHEA and the placebo groups [41]. In a prospective cohort study of 103 women with DOR, Hu et al. demonstrated that the DHEA and the control groups presented similar serum AMH levels [20]. Thus, more large-scale, well-designed studies are needed to clarify the effect of DHEA supplementation on serum AMH levels.

In addition to ovarian reserve, studies have shown that adjuvant treatment with DHEA ameliorates oocytes and embryos quality and subsequent IVF outcomes in women with DOR or PORs [17,42]. A network meta-analysis of randomized controlled trials (RCTs) conducted by Zhang et al. demonstrated that DHEA supplementation resulted in significantly higher clinical pregnancy rates as compared with a control group (odds ratio (OR) 2.46, 95% CI 1.16 to 5.23) in PORs [42]. Mitochondria plays a critical role in oocyte maturation, fertilization, embryo development, and pregnancy [43,44]. It has been reported that DHEA treatment improves mitochondrial function and reduces apoptosis of cumulus cells [45,46,47]. A prospective cohort study, on 38 PORs and 28 normal ovarian responders, showed that DHEA supplementation promoted mitochondrial mass, restored mitochondrial morphology, reduced mitochondrial fragmentation, increased mitochondrial fusion, and prevented mitophagy [47]. Moreover, increased IGF-1 levels have been shown to be associated with oocyte maturation, embryo development, and better IVF outcomes [48,49,50]. Taken together, DHEA supplementation may enhance both ovarian quantity and ovarian quality, resulting in better IVF outcomes. In this study, an age-specific normal reference range for serum DHEA-S levels was provided (Table 4). This table could help physicians to decide whether DHEA pretreatment was needed for infertile women. However, further large-scale, well-defined RCTs are still required to confirm the effectiveness of DHEA supplementation on IVF outcomes.

Several potential limitations should be highlighted when interpreting our results. First, the retrospective design poses a major limitation of this study. Second, a causal relationship could not be determined based on a cross-sectional study, which could be better examined in longitudinal studies. Thus, further longitudinal studies are required to verify the relationship. Third, our results may not be applicable to the general population since only infertile women were included in our study. Fourth, ethnicity in relation to serum AMH levels was not evaluated because all subjects included in our study were Taiwanese. Fifth, serum free T and sex hormone-binding globulin (SHBG) levels were not measured in our routine infertility survey.

In conclusion, our data demonstrated a positive association between serum DHEA-S and AMH levels in infertile women. Long-term longitudinal studies are needed to verify our results.

## Figures and Tables

**Table 1:** The characteristics of the study population.

	All Population (*n* = 2155)	Age < 35 Years (*n* = 972)	Age ≥ 35 Years (*n* = 1183)
Age (years)	35.1 ± 4.7	30.9 ± 2.7	38.6 ± 2.8
Body height (cm)	160.5 ± 5.8	160.8 ± 5.9	160.3 ± 5.7
Body weight (kg)	57.8 ± 10.5	56.8 ± 10.7	58.7 ± 10.4
BMI (kg/m^2^)	22.4 ± 3.8	21.9 ± 3.9	22.8 ± 3.8
TSH (μIU/mL)	1.7 ± 1.4	1.7 ± 1.7	1.7 ± 1.1
Prolactin (ng/mL)	15.5 ± 15.2	15.2 ± 12.0	15.7 ± 17.4
25(OH)vitamin D (ng/mL)	21.7 ± 6.9	21.0 ± 6.2	22.2 ± 7.3
FSH (mIU/mL)	5.4 ± 4.0	4.9 ± 3.0	5.8 ± 4.6
Testosterone (ng/mL)	0.35 ± 0.45	0.38 ± 0.48	0.32 ± 0.43
DHEA-S (μg/dL)	240.7 ± 113.6	262.7 ± 107.6	222.5 ± 115.3
AMH (ng/mL)	4.0 ± 4.0	5.2 ± 4.5	2.9 ± 3.1

Data are presented as mean ± standard deviation. BMI, body mass index; TSH, thyroid-stimulating hormone; FSH, follicle stimulation hormone; DHEA-S, dehydroepiandrosterone sulphate; AMH, anti-Müllerian hormone.

**Table 2:** Multiple linear regression analysis on the correlations between serum dehydroepiandrosterone sulphate (DHEA-S) and anti-Mullerian hormone (AMH) levels after adjustment for potential confounders.

Variables	All Women		Age < 35 Years		Age ≥ 35 Years	
	β	*p*	β	*p*	β	*p*
DHEA-S (μg/dL)	0.103	<0.001	0.113	0.004	0.091	0.009
Age (years)	−0.336	<0.001	−0.133	0.001	−0.334	<0.001
BMI (kg/m^2^)	0.047	0.059	0.058	0.131	0.035	0.314
FSH (mIU/mL)	−0.070	0.005	−0.076	0.045	−0.076	0.027
Prolactin (ng/mL)	−0.034	0.172	−0.061	0.112	−0.022	0.521

BMI, body mass index; FSH, follicle stimulation hormone.

**Table 3:** The characteristics of the study population divided based on serum DHEA-S quartile categories.

	Quartile of Serum DHEA-S				
	Q1 (*n* = 502)	Q2 (*n* = 523)	Q3 (*n* = 523)	Q4 (*n* = 513)	*p*-Value for Trends
DHEA-S (μg/dL)	≤168.05	168.05~221.0	221.0~292.95	≥292.95	
Age (years)	36.8 ± 4.5 *	35.8 ± 4.5 *	34.4 ± 4.5 *	33.4 ± 4.7	<0.001
Body height (cm)	160.6 ± 5.5	160.8 ± 5.5 *	160.3 ± 5.7	159.8 ± 5.4	0.018
Body weight (kg)	57.4 ± 10.4	57.7 ± 10.3	57.3 ± 10.0	57.9 ± 10.9	0.669
BMI (kg/m^2^)	22.3 ± 3.9	22.3 ± 3.8	22.3 ± 3.8	22.6 ± 4.1	0.161
TSH (μIU/mL)	1.7 ± 1.5	1.6± 1.0	1.6 ± 1.0	1.7 ± 1.9	0.931
Prolactin (ng/mL)	14.5 ± 13.4	15.0 ± 11.7	15.8 ± 10.9	16.5 ± 22.1	0.025
Vitamin D (ng/mL)	21.6 ± 7.3	21.4 ± 6.9	21.7 ± 6.5	22.3 ± 6.9	0.192
FSH (mIU/mL)	5.7 ± 4.2 *	5.7 ± 4.8 *	5.0 ± 3.2	5.0 ± 2.7	<0.001
Testosterone (ng/mL)	0.23 ± 0.10 *	0.28 ± 0.14 *	0.32 ± 0.13 *	0.37 ± 0.15	<0.001
AMH (ng/mL)	2.9 ± 2.9 *	3.8 ± 3.7 *	4.3 ± 3.8	4.8 ± 5.0	<0.001

Data are presented as mean ± standard deviation; *p*-values for trends were generated by linear regression analysis; * statistically significantly different from the highest quartile category (Q4) using Bonferroni’s method in an analysis of variance (ANOVA) test. DHEA-S, dehydroepiandrosterone sulphate; BMI, body mass index; TSH, thyroid-stimulating hormone; Vitamin D, 25(OH)Vitamin D; FSH, follicle stimulation hormone; AMH, anti-Müllerian hormone.

**Table 4:** Serum DHEA-S level distribution (μg/dL) according to age.

Age (Years)	Mean	Percentile					*n*
		5th	10th	Median	90th	95th	
20~25	291.7	117.7	157.3	272.3	470.1	541.5	38
26~30	280.2	139.9	158.8	264.5	412.6	476.0	310
31~35	248.1	109.0	134.8	234.2	370.4	423.5	753
36~40	226.7	94.6	115.1	206.1	354.0	432.2	682
41~46	204.1	80.8	104.6	181.4	314.6	367.3	278
All	240.7	102.8	125.1	221.0	370.4	436.8	2061

DHEA-S, dehydroepiandrosterone sulphate.

## Data Availability

The data presented in this study can be available on request from the corresponding author.
